# A Heterocyclic Polyurethane with Enhanced Self-Healing Efficiency and Outstanding Recovery of Mechanical Properties

**DOI:** 10.3390/polym12040968

**Published:** 2020-04-21

**Authors:** Jinsil Kim, Pyong Hwa Hong, Kiwon Choi, Gyeongmin Moon, Jungsoon Kang, Seoyun Lee, Sungkoo Lee, Hyun Wook Jung, Min Jae Ko, Sung Woo Hong

**Affiliations:** 1 Intelligent Sustainable Materials R&D Group, Korea Institute of Industrial Technology, 89 Yangdaegiro-gil, Ipjang-myeon, Seobuk-gu, Cheonan-si, Chungcheongnam-do 31056, Korea; kjs2761@kitech.re.kr (J.K.); hph0302@kitech.re.kr (P.H.H.); tactfully@kitech.re.kr (G.M.); sungkoo@kitech.re.kr (S.L.); 2 Department of Chemical and Biological Engineering, Korea University, 145 Anam-ro, Seongbuk-gu, Seoul 02841, Korea; hwjung89@korea.ac.kr; 3 Department of Chemical Engineering, Hanyang University, 222 Wangsimni-ro, Seongdong-gu, Seoul 04763, Korea; laniah304@naver.com (K.C.); 099kjs@naver.com (J.K.); seoyunii@hanyang.ac.kr (S.L.); 4 Institute of Nano Science and Technology, Hanyang University, 222 Wangsimni-ro, Seongdong-gu, Seoul 04763, Korea

**Keywords:** self-healing, polyurethane, hydrogen bonding interaction, heterocyclic, flexible display

## Abstract

A functional polyurethane based on the heterocyclic group was synthesized and its self-healing and mechanical properties were examined. To synthesize a heterocyclic polyurethane, a polyol and a heterocyclic compound with di-hydroxyl groups at both ends were blended and the blended solution was reacted with a crosslinker containing multiple isocyanate groups. The heterocyclic polyurethane demonstrates better self-healing efficiency than the conventional polyurethane with no heterocyclic groups. Furthermore, unlike the conventional self-healing materials, the heterocyclic polyurethane examined in this study shows an outstanding recovery of the mechanical properties after the self-healing process. These results are attributed to the unique supramolecular network resulting from the strong hydrogen bonding interaction between the urethane group and the heterocyclic group in the heterocyclic polyurethane matrix.

## 1. Introduction

As display technology has evolved from flat displays to flexible (foldable, rollable, wearable, or stretchable) displays [1,2,3,4,5,6], the demand for new functional materials for flexible displays has increased significantly, particularly for surface protection. While hard coating materials have been extensively used to protect a flat display surface from mechanical surface damage such as dents or scratches (linear tearing of the surface), the properties of these coatings prevent their adaptation for use in flexible displays. For example, during folding, the inside surface of the folded display will be compressed while the outside surface will be extended. Because a hard coating layer is usually located at the outermost layer of the display, repeated folding and unfolding will generate significant stress accumulation on the hard coating layer. As conventional hard coating materials are known to be fragile and easily broken by mechanical stress, this cumulative stress will cause severe mechanical damage to the display surface and therefore will decrease the life expectancy of the displays. Self-healing polymeric materials are an emerging class of smart functional materials and have been widely studied during the past several decades [7]. While they are more susceptible to damage caused by mechanical stress because of their low mechanical surface properties, these materials can effectively self-heal the areas damaged by the mechanical stress. In light of the critical issues mentioned above, self-healable polymeric coating materials will be preferred to hard coating materials for application in flexible displays. Therefore, endowing conventional polymeric materials with the ability to self-heal both dents and scratches is considered to be vital for the development of next-generation flexible displays [8,9].

Traditionally, both extrinsic and intrinsic strategies have been employed to introduce the self-healing capability to polymeric materials. Extrinsic systems depend on embedded capsules or vascular networks [10,11,12]. When the material is damaged, the embedded container will release the contained healing agents to heal the damage by polymerization or chemical reaction. However, repeated healing at the same site is inherently difficult due to the limited amount of the healing agent inside the container, and the healing ability will disappear immediately after the healing agent is depleted [7]. By contrast, intrinsic systems utilize the reversible interaction between the components of the matrix [13,14,15,16], allowing them to repeatedly self-heal the same damaged areas. It is also noted that in contrast to intrinsic systems, it is difficult for the extrinsic systems to maintain the initial optical properties after the self-healing process because of the large difference in the color and refractive index between the intact matrix and the healed region. For these reasons, it is concluded that intrinsic systems are a more appropriate solution for applications, such as in display technologies, that require excellent optical properties.

Polyurethane is a widely used intrinsic self-healing material that has been extensively studied because it can effectively recover from the dents on the surface due to its high elasticity [17,18,19,20,21]. However, while polyurethane can self-heal the dents on its surface, it cannot fully heal the scratch damage areas due to the lack of a strong specific interaction between the polyurethane chains. Moreover, similar to the conventional self-healable materials, polyurethane also faces the traditional trade-off between mechanical strength and self-healing capacity that will result in a poor recovery of the mechanical properties after damage.

In this study, we developed a new self-healable polyurethane containing a functional heterocyclic moiety. Additionally, the self-healing and mechanical properties of the new self-healable polyurethane were examined in terms of the supramolecular interactions between the urethane group and the heterocyclic group. Compared to the conventional polyurethane, the heterocyclic polyurethane proposed in this study demonstrates superior self-healing performance. Moreover, it also shows excellent recovery of mechanical properties after the self-healing process, effectively eliminating the trade-off between self-healing and the deterioration of mechanical properties present for the conventional self-healing materials.

## 2. Materials and Methods

### 2.1. Materials

*N,N*-Dimethylacetamide (DMAc) (Alfa-Aesar, 99.8%, Haverhill, MA, USA), ethyl-*N*-methylcabamate (U) (Tokyo Chemical Industry Co., Ltd., >98%, Tokyo, Japan), tetrahydrofuran (T) (Alfa-Aesar, 99.8%, Haverhill, MA, USA), CHCI_3_ (Sigma Aldrich, ≥99%, St. Louis, MI, USA), 1,3,5-tris(6-isocyanatohexyl)-1,3,5-triazinane-2,4,6-trione (HDIt) (Asahi Kasei, 98%, Tokyo, Japan), 2,5-bishydroxymethyl tetrahydrofuran (BHMTHF) (Hangzhou Chempro Tech Co. Ltd., ≥98%, Hangzhou, China), 1,4-benzenedimethanol (BHMB) (Tokyo Chemical Industry Co., Ltd., >99%, Tokyo, Japan), 1,2-dihydroxyethane (DHE) (Tokyo Chemical Industry Co., Ltd., >99.5%, Tokyo, Japan), and diethyl ether (E) (Tokyo Chemical Industry Co., Ltd., >99.5%, Tokyo, Japan) were used as received. 2,2′-Azobis(2-methylpropionitrile) (Daejungchem, 99%, Siheung, Korea) was recrystallized before use, and 2-hydroxyethyl methacrylate (HEMA) (Tokyo Chemical Industry Co., Ltd., >99%, Tokyo, Japan), and butyl methacrylate (BMA) (Tokyo Chemical Industry Co., Ltd., >99%, Tokyo, Japan) were passed through a basic alumina column prior to use.

### 2.2. Synthesis of Polyol (Poly(2-hydroxyl methacrylate-r-butyl methacrylate) (poly(HEMA-r-BMA))

BMA (40.163 g, 0.282 mol), HEMA (10.041 g, 0.077 mol), AIBN (1.178 g, 0.007 mol), and DMAc (51.381 g) were added to a 3-neck round bottom flask purged with nitrogen gas, and the solution was vigorously stirred at 70 °C for 12 h. The final polyol solution appeared as a transparent oil with the concentration of 50 wt.% and was used without further purification. *M*_n_ = 12,000; PDI = 2.12.

### 2.3. Preparation Conventional Polyurethane (PU), PU with Aromatic Moiety (PUB), PU with Heterocyclic Moiety (PUT), and PU with Aliphatic Moiety (PUD)

All of the solutions were homogeneously mixed and degassed using a paste mixer prior to coating. A flow-coating technique was employed to prepare all of the samples in this study. To prepare PU, a polyol solution in DMAc (50 wt.%, 3.66 g) and a crosslinker (HDIt) solution in DMAc (50 wt.%, 1 g) were blended, and the resulting mixed solution was coated onto the substrate and heated at 60 °C for 0.5 h and then at 170 °C for 1 h. To prepare PUB, a polyol solution in DMAc (50 wt.%, 2.77 g), BHMB solution in DMAc (20 wt.%, 0.24 g), and crosslinker (HDIt) solution in DMAc (50 wt.%, 1 g) were blended, and the resulting mixed solution was coated onto the substrate and heated at 60 °C for 0.5 h and then at 170 °C for 1 h. To prepare PUT, a polyol solution in DMAc (50 wt.%, 2.77 g), BHMTHF solution in DMAc (19.13 wt.%, 0.24 g), and crosslinker (HDIt) solution in DMAc (50 wt.%, 1 g) were blended, and the resulting mixed solution was coated onto the substrate and heated at 60 °C for 0.5 h and then at 170 °C for 1 h. To prepare PUD, a polyol solution in DMAc (50 wt.%, 2.77 g), DHE solution in DMAc (8.98 wt.%, 0.24 g), and crosslinker (HDIt) solution in DMAc (50 wt.%, 1 g) were blended, and the resulting mixed solution was coated onto the substrate and heated at 60 °C for 0.5 h and then at 170 °C for 1 h.

### 2.4. Characterization

The optical properties of the samples were measured using a spectrophotometer (CM-3600d, Konica Minolta, Tokyo, Japan). The thermal properties were analyzed by differential scanning calorimetry (DSC) (DSC Q20, TA Instrument, New Castle, DE, USA) with a heating rate of 10 °C/min under nitrogen atmosphere. The structural analysis was performed using ^1^H nuclear magnetic resonance (NMR) spectroscopy (VNMRS 600 MHz, Varian, Crawley, UK) with CDCl_3_ as the NMR solvent, gel permeation chromatography (GPC) (Waters GPC system, Waters, Milford, MA, USA) equipped with a refractive index detector using tetrahydrofuran as the eluent with the columns calibrated against the standard polystyrene samples, and Fourier-transform infrared spectroscopy (FT-IR) (Nicolet iS50, Thermo Fisher Scientific, Waltham, MA, USA) using a spectrometer equipped with attenuated total reflectance accessory. The self-healing properties were measured using a nanoscratch tester (NST^3^, Anton paar, Graz, Austria) equipped with a conical tip with the size of 20 μm and a white light interferometer (NewView^TM^, ZYGO, Berwyn, PA, USA) at a loading speed of 2 mm/min. The mechanical properties were measured using a universal tensile machine (QM100SE, QMESYS, Gyeonggi, Korea) at a rate of 10 mm/min and a cell load of 10 kN. All of the specimens were prepared according to ASTM D1708: dog-bone in shape; 38 mm × 15 mm overall in size; 5 mm × 22 mm in the gauge area.

## 3. Results and Discussion

Table 1 shows the designations and chemical structures of the samples in this study (see Figure S1 in the Supplementary Information for the overall synthetic route, ^1^H nuclear magnetic resonance (NMR) spectrum, and gel permeation chromatography (GPC) results for polyol). As a reference, conventional polyurethane (PU) was prepared by reacting a polyol solution and a crosslinker solution. A series of self-healable polyurethanes (PUB, PUT, and PUD) were prepared by reacting a polyol solution, crosslinker solution, and functional diol solution where 1,4-benzenedimethanol (BHMB), 2,5-bishydroxymethyl tetrahydrofuran (BHMTHF), and 1,2-dihydroxyethane (DHE) were used as the aromatic functional diol, heterocyclic functional diol, and aliphatic functional diol, respectively. It is noted that all of the functional diol solutions have identical OH values, and the theoretical degree of crosslinking of all of the samples was set to 100%. The thermal and optical properties were analyzed using a differential scanning calorimeter (DSC), a thermal gravimetric analyzer (TGA), and a spectrophotometer (see Figure S2–S4 in the Supplementary Information for the DSC thermograms and TGA curves and the detailed optical properties of PU, PUB, PUT, and PUD). Comparison of PU to PUB, PUT, and PUD shows that all of the samples have similar transition temperatures and also show high transmittance and low haze and yellow index values, indicating that all of the functional diols used in this study did not significantly affect the initial thermal and optical properties of PU as listed in Table 2. It is generally known that a residual solvent in polymeric system can act as a plasticizer and significantly affect the final glass transition temperature. When the TGA results were analyzed to measure the amount of residual solvent, all of the TGA curves showed no observable change in weight, which indicates that all of the samples used in this study have no residual solvents.

**Figure 1:**
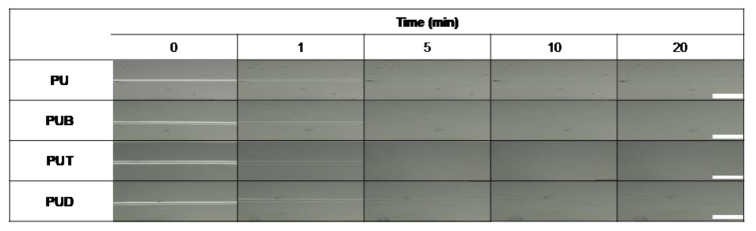
Optical microscope images of PU, PUB, PUT, and PUD before and after the self-healing process. The loading force of 3 N was equally applied to all the samples and the self-healing temperature was set to be *T*_g_ + 10 °C. The scale bar is 100 µm.

**Figure 2:**
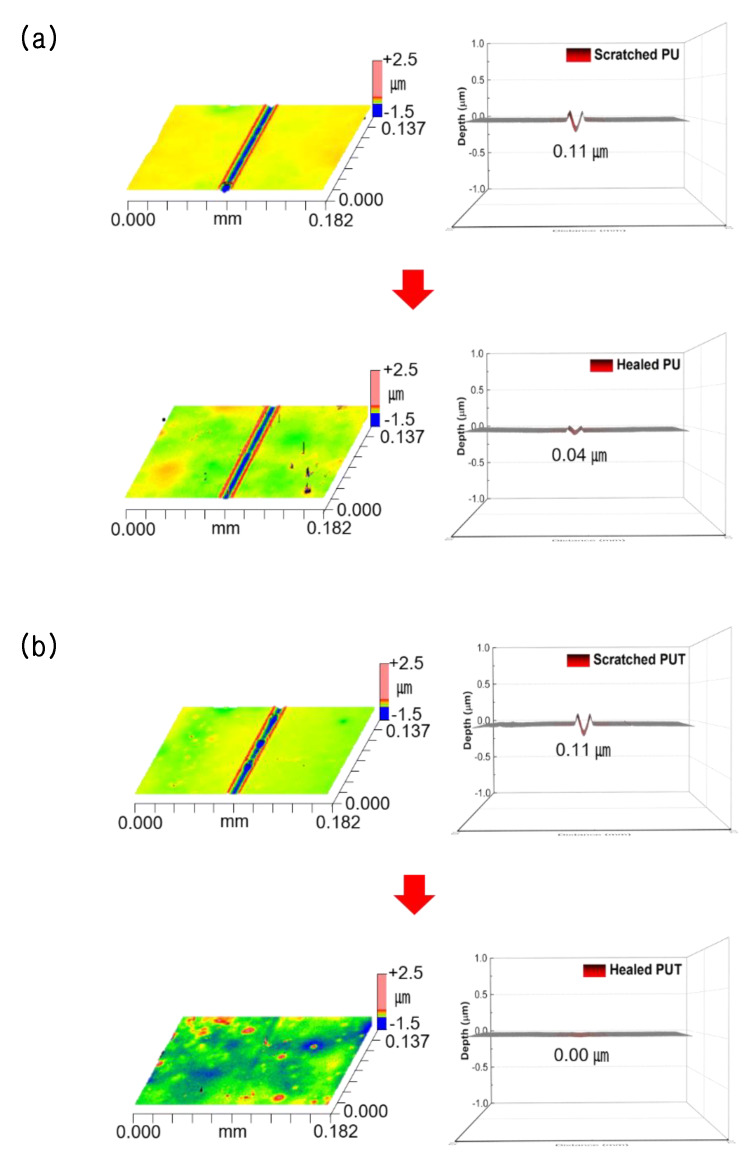
Interferometer images of (**a**) PU and (**b**) PUT before and after the self-healing process. The loading force of 50 mN was applied to both samples and the self-healing temperature and time were set to *T*_g_ + 10 °C and 10 min, respectively.

A self-healing experiment was conducted for PU, PUB, PUT, and PUD using the single scratch technique. Figure 1 shows the optical microscopy images before and after the self-healing process. It should be noted that PUB, PUT, and PUD contain an aromatic benzene moiety, heterocyclic tetrahydrofuran moiety, and aliphatic ethylene moiety, respectively, where all of these moieties have approximately the same molecular size. Interestingly, while PU, PUB, and PUD still retained traces of damage even after the self-healing process, PUT successfully self-healed the damaged area and recovered to its initial state within a short time. To quantitatively evaluate the self-healing efficiency of PU and PUT, a nano/micro scratching technique was used. As shown in Figure 2, PUT shows perfect self-healing performance (0.1–0 μm, 100%) in contrast to PU (0.11–0.4 μm, 64%), indicating that the heterocyclic group can provide an outstanding self-healing capability in conventional polyurethanes.

**Figure 3:**
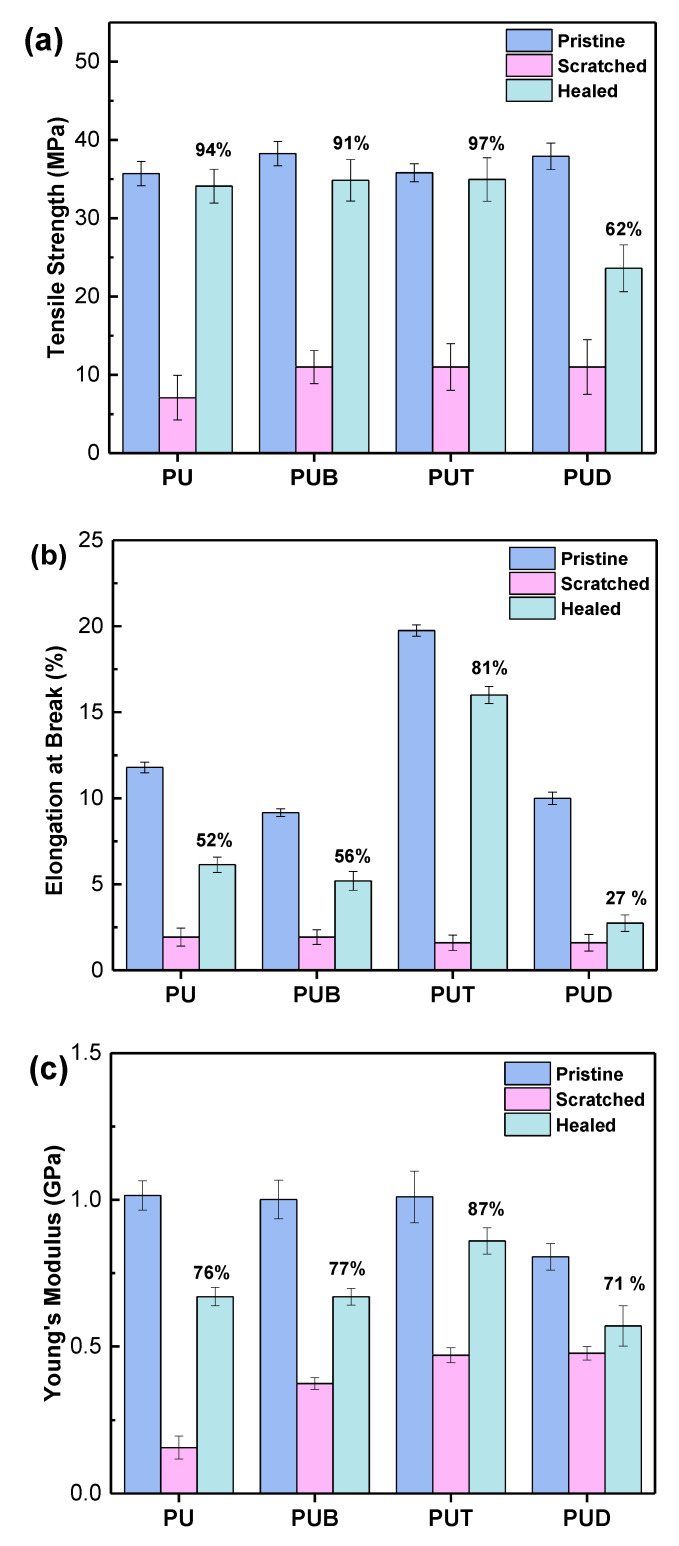
(**a**) Tensile strength, (**b**) elongation at break, and (**c**) Young’s modulus of PU, PUB, PUT, and PUD before and after the self-healing process. All data points were obtained by averaging the results of five measurements.

Figure 3 demonstrates an additional and crucial advantage of the introduction of the heterocyclic group to conventional polyurethanes. Various mechanical properties before and after the self-healing process were measured using a universal testing machine. PUT shows the best recovery of tensile strength, elongation at break, and Young’s modulus compared to PU, PUB, and PUD. Considering the fact that all of the functional diol solutions have identical OH values and all of the functional diols have approximately the same molecular size, it is concluded that the heterocyclic group can also endow conventional polyurethane with superior recovery of mechanical properties. 

**Figure 4:**
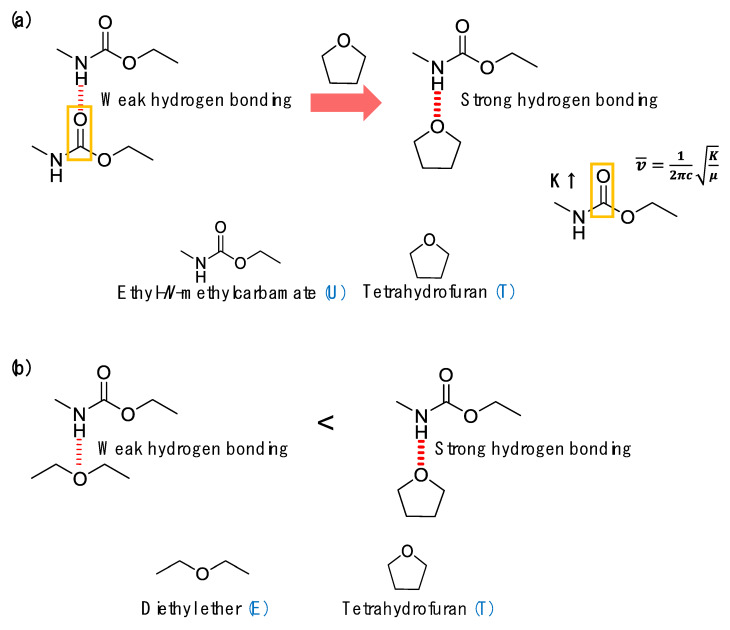
Schematic illustrations of: (**a**) the hydrogen bonding between two urethane groups (Us) and the hydrogen bonding between the urethane group (U) and the heterocyclic group (T) and (**b**) the hydrogen bonding between the urethane group (U) and the heterolinear group (E) and that between the urethane group (U) and the heterocyclic group (T).

**Figure 5:**
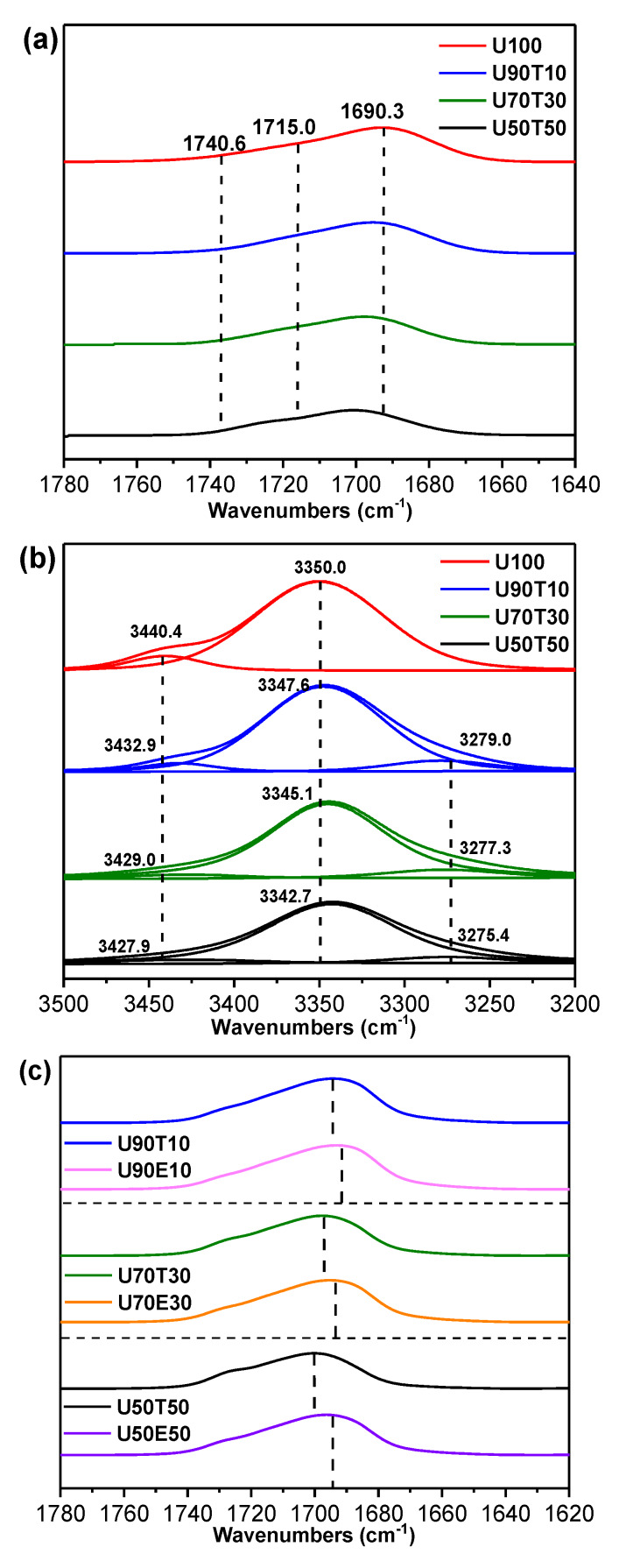
(**a**) The C=O stretching regions of the U and UT blends, (**b**) the N-H stretching regions of the U and UT blends, and (**c**) the C=O stretching regions of the UT blends and UE blends in the FT-IR spectra. Each spectrum in (**b**) was resolved into its components by the curve-fitting method.

To interpret these phenomena, a model system was used to propose the mechanism associated with improved self-healing performance and enhanced recovery of mechanical properties. Ethyl-*N*-methylcarbamate (U), tetrahydrofuran (T), and diethyl ether (E) were used to imitate PU, the heterocyclic group, and the heterolinear group, respectively. Figure 4 and Table 3 show the chemical structures of U, T, and E and the designations and compositions of the samples used for the model system. Based on previous reports [22], it is known that urethane forms three different types of hydrogen bonds (free (noN–Hydrogen bonded), single-hydrogen bonds, and double-hydrogen bonds) [21,23]. As shown in Figure 5a, the FT-IR spectrum of U100 (pure U) showed the double-hydrogen bonded C=O band at 1690.3 cm^−1^, the single-hydrogen bonded C=O band (about 1715.0 cm^−1^) as a shoulder, and the free C=O band (about 1740.6 cm^−1^) as a weak shoulder (barely detectable). Interestingly, the peaks corresponding to the double- and single-hydrogen bonding both shifted to higher wavenumbers with increasing fraction of T, as shown in Figure 5a and Table 3. This can be explained as follows. As graphically shown in Figure 4a, when T approaches two U molecules that are connected by the weak hydrogen bonding between C=O and N–H, T will break the existing hydrogen bonds between the two U molecules due to the stronger specific interaction between the –O– in T and N–H in U. This will result in a stronger hydrogen bonding interaction between T and U and the force constant (K) of the dropped U will increase because the unpaired electrons in the C=O of the dropped U no longer interact with the partially positively charged hydrogen atom in N–H [20,24,25,26,27,28,29,30]. As a result, based on the spring model for FT-IR spectroscopy, all of the peaks associated with the hydrogen bonded C=O shifted to higher wavenumbers as shown in Figure 5a.

To analyze the FT-IR results quantitatively, the N–H bands were deconvoluted by fitting with mixed Lorentzian–Gaussian shaped components as shown in Figure 5b. The U100 spectrum initially contained the band of free N–H at 3440.4 cm^−1^ and the band of the N–H hydrogen bonded to U at 3350.0 cm^−1^ as listed in Table 3. When T was added to U, a new peak associated with the band of the N–H hydrogen bonded to T appeared due to the formation of stronger hydrogen bonding between U and T compared to that between two U molecules. As the weight fraction of T further increased, the peak position corresponding to the new peak shifted to lower wavenumbers. In addition, the fitted peak area of the N–H hydrogen bonded with T increased and that of the N–H hydrogen bonded with U decreased simultaneously as listed in Table 3. All of these results clearly demonstrate that the heterocyclic group significantly enhances the hydrogen bonding interaction in conventional polyurethanes.

**Figure 6:**
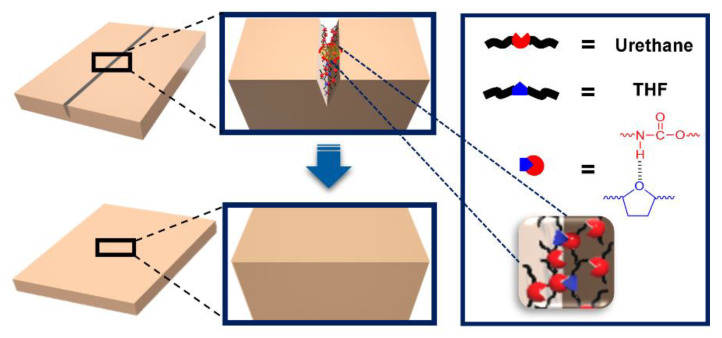
Schematic illustration of the self-healing mechanism of PUT. Red and blue represent the urethane group and heterocyclic group, respectively.

Lastly, the effect of the chemical structure of the functional diols containing heteroatoms on the resulting hydrogen bonding interaction was examined. Comparison of the FT-IR spectra of the blend of U and heterocyclic T to those of U and heterolinear E shows that the peak position of the double-hydrogen bonded C=O of U90T10 appeared at 1694.3 cm^−1^, while that of U90E10 appeared at 1693.2 cm^−1^ as shown in Table 3 and Figure 5c. The difference between the corresponding peak positions of the UT blend and that of the UE blend increased as the amount of the heterofunctional diol increased as listed in Table 3. Considering the fact that both T and E have approximately the same molecular weight, it is concluded that the hydrogen bonding between U and T is much stronger than that between U and E. It is noted that crosslinked polyurethane is generally known to have structural irregularity. Interestingly, from the FT-IR results of PU, the areal ratio of hydrogen-bonded carbonyl groups to free carbonyl groups is 88.7 to 11.3, which implies that most urethane groups in crosslinked polyurethane are also connected by hydrogen bonding (see Figure S5 in the Supplementary Information for the N–H stretching region of PU). Based on all of the FT-IR results derived from the model system, it is strongly suggested that the heterocyclic group can significantly enhance the hydrogen bonding interaction in conventional polyurethane, resulting in a unique supramolecular network structure that can generate the main driving force to significantly improve both self-healing and mechanical properties as graphically shown in Figure 6.

## 4. Conclusions

In summary, a new self-healable polyurethane based on the heterocyclic group was prepared in this study. In contrast to conventional polyurethane, the heterocyclic self-healing polyurethane shows significantly enhanced self-healing performances. Furthermore, the heterocyclic self-healing polyurethane proposed in this study can overcome the traditional trade-off between self-healing and the deterioration of mechanical properties. This work can provide an avenue for the development of new functional materials for next-generation flexible displays.

## Figures and Tables

**Table 1:** Chemical structures of polyol, crosslinker, and functional diols and the designations and compositions of the samples.

Sample Designations	Compositions		
	Polyol	Crosslinker (HDIt)	Functional Diols
PU	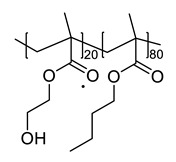	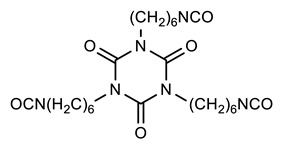	None
PUB			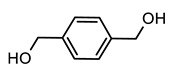 BHMB
PUT			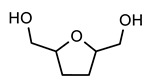 BHMTHF
PUD			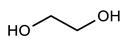 DHE

**Table 2:** Optical properties (transmittance (*T*_r_), yellow index (YI), and haze), thermal property (glass transition temperature (*T*_g_)), and thicknesses of conventional polyurethane (PU), PU with aromatic moiety (PUB), PU with heterocyclic moiety (PUT), and PU with aliphatic moiety (PUD). All of the data points were obtained by averaging the results of three measurements.

Sample Designations	Optical Properties			Thermal Property	Thickness (µm)
	*T*_r_ (%)	YI	Haze	*T*_g_ (°C)	
PU	91.7 ± 0.12	0.05 ± 0.02	0.2 ± 0.03	58.0	12.5 ± 0.23
PUB	91.6 ± 0.15	0.02 ± 0.01	0.3 ± 0.02	60.0	11.2 ± 0.33
PUT	91.7 ± 0.14	0.05 ± 0.01	0.1 ± 0.04	57.2	10.9 ± 0.21
PUD	91.7 ± 0.21	0.06 ± 0.03	0.2 ± 0.02	59.1	10.0 ± 0.13

**Table 3:** Designations and compositions of the samples used in the model system and the characteristics of the N–H and C=O bands in the FT-IR spectra. U, T, and E stand for ethyl-*N*-methylcarbamate, tetrahydrofuran, and diethyl ether, respectively.

Sample Designations	Compositions (wt.%)			Free N–H		Hydrogen Bonded N–H (to U)		Hydrogen Bonded N–H (to T)		Hydrogen Bonded C=O
	U	CHCl_3_	T	Wave Number (cm^−1^)	Area (%)	Wave Number (cm^−1^)	Area (%)	Wave Number (cm^−1^)	Area (%)	Wave Number (cm^−1^)
		Solvent for U								
U100	70	30	0	3440.4	8.5	3350.0	91.5	-	-	1690.3
U90T10	63	31	6	3432.9	5.4	3347.8	85.4	3279.0	9.2	1694.3
U70T30	49	36	15	3429.0	6.0	3345.1	85.0	3277.3	9.7	1697.7
U50T50	35	47	18	3427.9	6.6	3342.7	82.5	3275.4	10.9	1700.2
U90E10	63	31	6	-	-	-	-	-	-	1693.2
U70E30	49	36	15	-	-	-	-	-	-	1695.3
U50E50	35	47	18	-	-	-	-	-	-	1696.4

## Supplementary Materials

The following are available online at https://www.mdpi.com/2073-4360/12/4/968/s1, Figure S1: (a) Overall scheme for synthesis and ^1^H NMR spectrum in CDCl_3_ and (b) GPC trace of polyol (poly(HEMA-r-BMA)), Figure S2: DSC thermograms of PU, PUB, PUT, and PUD, Figure S3: Optical properties of PU, PUB, PUT, and PUD: (a) transmittance; (b) haze and yellow index. All data points were obtained by averaging three measurements, Figure S4: TGA curves of PU, PUB, PUT, and PUD, Figure S5: The N-H stretching region of PU in the FT-IR spectra. The spectrum was resolved into its components by the curve-fitting method.

## References

[1] Lai Y., Kuang X., Zhu P., Huang M.M., Dong X., Wang D.J. (2018). Colorless, Transparent, Robust, and Fast Scratch-Self-Healing Elastomers via a Phase-Locked Dynamic Bonds Design. Adv. Mater..

[2] Dou Y.B., Zhou A., Pan T., Han J.B., Wei M., Evans D.G., Duan X. (2014). Humidity-triggered self-healing films with excellent oxygen barrier performance. Chem. Commun..

[3] Benight S.J., Wang C., Tok J.B.H., Bao Z.A. (2013). Stretchable and self-healing polymers and devices for electronic skin. Prog. Polym. Sci..

[4] Guo Y., Zheng K.Q., Wan P.B. (2018). A Flexible Stretchable Hydrogel Electrolyte for Healable All-in-One Configured Supercapacitors. Small.

[5] Huang L., Yi N., Wu Y., Zhang Y., Zhang Q., Huang Y., Ma Y., Chen Y. (2013). Multichannel and repeatable self-healing of mechanical enhanced graphene-thermoplastic polyurethane composites. Adv. Mater..

[6] Huynh T.P., Sonar P., Haick H. (2017). Advanced Materials for Use in Soft Self-Healing Devices. Adv. Mater..

[7] Hager M.D., Greil P., Leyens C., van der Zwaag S., Schubert U.S. (2010). Self-healing materials. Adv. Mater..

[8] Nathan A., Ahnood A., Cole M.T., Lee S., Suzuki Y., Hiralal P., Bonaccorso F., Hasan T., Garcia-Gancedo L., Dyadyusha A. (2012). Flexible Electronic: The Next Ubiquitous Platform. Proc. IEEE.

[9] Fischer H. (2010). Self-repairing material system a dream or a reality. Nat. Sci..

[10] Rogers J.A., Bao Z., Baldwin K., Dodabalapur A., Crone B., Raju V., Kuck V., Katz H., Amundson K., Ewing P. (2001). Paper-like electronic displays: Large-area rubber-stamped plastic sheets of electronics and microencapsulated electrophoretic inks. Proc. Natl. Acad. Sci. USA.

[11] Brown E., White S., Sottos N. (2005). Retardation and repair of fatigue cracks in a microcapsule toughened epoxy composite-Part II: In situ self-healing. Compos. Sci. Technol..

[12] Toohey K.S., Sottos N.R., Lewis J.A., Moore J.S., White S.R. (2007). Self-healing materials with microvascular networks. Nat. Mater..

[13] Blaiszik B.J., Kramer S.L., Olugebefola S.C., Moore J.S., Sottos N.R., White S.R. (2010). Self-Healing Polymers and Composites. Annu. Rev. Mater. Res..

[14] Yuan Y.C., Yin T., Rong M.Z., Zhang M.Q. (2008). Self-healing in polymers and polymer composites. Concepts, realization and outlook: A review. Express Polym. Lett..

[15] Guoqiang L., Harper M. (2005). Recent Advances in Smart Self-Healing Polymers and Composites.

[16] Nathalie K.G., Kim K., Oehlenschlaeger J.Z., Stefan H., Friedrich G.S., Christopher B.K. (2012). Current Trends in the Field of Self-Healing Materials. Macromol. Chem. Phys..

[17] Herck V.N., Du Prez F.E. (2018). Fast Healing of Polyurethane Thermosets Using Reversible Triazolinedione Chemistry and Shape-Memory. Macromolecules.

[18] Du P., Wu M., Liu X., Zheng Z., Wang X., Sun P., Joncheray T., Zhang Y. (2014). Synthesis of linear polyurethane bearing pendant furan and cross-linked healable polyurethane containing Diels–Alder bonds. New J. Chem..

[19] Saraf V.P., Glasser W.G. (1984). Engineering plastics from lignin. Structure property relationships in solution cast polyurethane films. J. Appl. Polym. Sci..

[20] Mailhot B., Komvopoulos K., Ward B., Tian Y., Somarjai G. (2001). Mechanical and friction properties of thermoplastic polyurethanes determined by scanning force microscopy. J. Appl. Phys..

[21] Sonnenschein M.F. (2014). Polyurethanes: Science, Technology, Markets, and Trends.

[22] Lee S., Hong P.H., Kim J., Choi K., Moon G., Kang J., Lee S., Ahn J.B., Eom W., Ko M.J. (2020). Highly Self-Healable Polymeric Blend Synthesized Using Polymeric Glue with Outstanding Mechanical Properties. Macromolecules.

[23] Randall D., Lee S. (2002). The Polyurethanes Book.

[24] Mattia J., Painter P. (2007). A comparison of hydrogen bonding and order in a polyurethane and poly(urethane-urea) and their blends with poly(ethylene glycol). Macromolecules.

[25] Huacuja-Sań chez J., Muller K., Possart W. (2016). Water diffusion in a crosslinked polyether-based polyurethane adhesive. Int. J. Adhes. Adhes..

[26] Lee S.H., Wang K.Y., Hsu L.S. (1987). Spectroscopic analysis of phase separation behavior of model polyurethanes. Macromolecules.

[27] Coleman M.M., Skrovanek J.D., Hu J., Painter C.P. (1988). Hydrogen bonding in polymer blends. 1. FTIR studies of urethane-ether blends. Macromolecules.

[28] Skrovanek D.J., Howe S.E., Painter P.C., Coleman M.M. (1985). Hydrogen bonding in polymer: Infrared temperature studies of an amorphous polyamide. Macromolecules.

[29] Socrates G. (2001). Infrared and Raman Characteristic Group Frenquencies.

[30] Fessenden R.J., Fessenden J.S. (1993). Organic Chemistry.

