# Supplementary figures and images for: A Heterocyclic Polyurethane with Enhanced Self-Healing Efficiency and Outstanding Recovery of Mechanical Properties
Source: Polymers (Basel). 2020 Apr 21;12(4):968. doi: 10.3390/polym12040968 (PMC7240572; doi:10.3390/polym12040968)

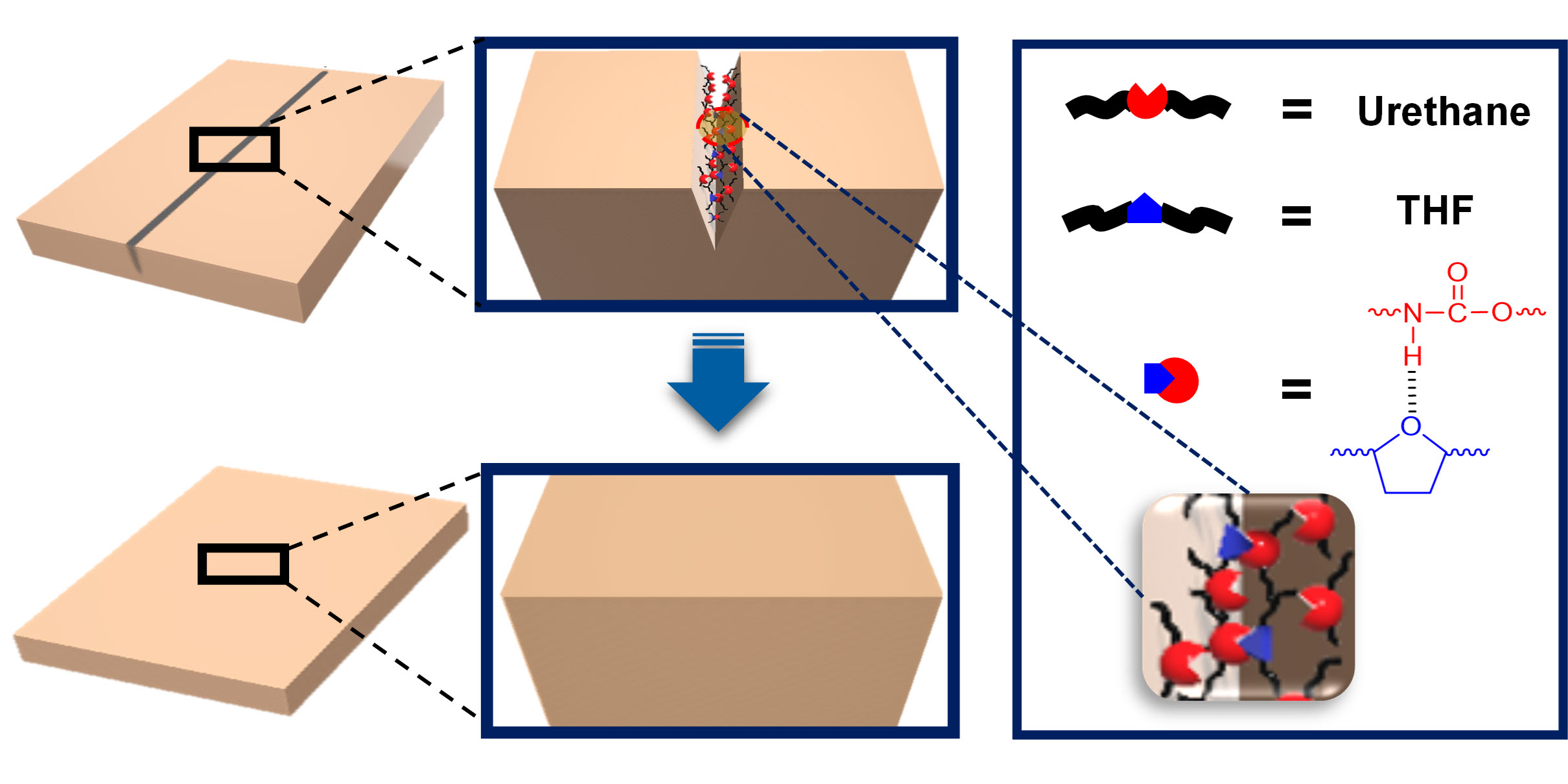

Supplement: Supplementary file 1 [file polymers-12-00968-s001.jpg]
